# Digitized Spiral Drawing: A Possible Biomarker for Early Parkinson’s Disease

**DOI:** 10.1371/journal.pone.0162799

**Published:** 2016-10-12

**Authors:** Marta San Luciano, Cuiling Wang, Roberto A. Ortega, Qiping Yu, Sarah Boschung, Jeannie Soto-Valencia, Susan B. Bressman, Richard B. Lipton, Seth Pullman, Rachel Saunders-Pullman

**Affiliations:** 1 Department of Neurology, Mount Sinai Beth Israel Medical Center, New York, New York, United States of America; 2 Department of Neurology, University of California San Francisco, San Francisco, California, United States of America; 3 Department of Epidemiology and Population Health, Albert Einstein College of Medicine Bronx, New York, New York, United States of America; 4 Department of Neurology, Columbia University, New York, New York, United States of America; 5 Department of Neurology, Albert Einstein College of Medicine Bronx, New York, New York, United States of America; 6 Department of Neurology, Icahn School of Medicine at Mount Sinai, New York, New York, United States of America; Institute of Health Science, CHINA

## Abstract

**Introduction:**

Pre-clinical markers of Parkinson’s Disease (PD) are needed, and to be relevant in pre-clinical disease, they should be quantifiably abnormal in early disease as well. Handwriting is impaired early in PD and can be evaluated using computerized analysis of drawn spirals, capturing kinematic, dynamic, and spatial abnormalities and calculating indices that quantify motor performance and disability. Digitized spiral drawing correlates with motor scores and may be more sensitive in detecting early changes than subjective ratings. However, whether changes in spiral drawing are abnormal compared with controls and whether changes are detected in early PD are unknown.

**Methods:**

138 PD subjects (50 with early PD) and 150 controls drew spirals on a digitizing tablet, generating x, y, z (pressure) data-coordinates and time. Derived indices corresponded to overall spiral execution (severity), shape and kinematic irregularity (second order smoothness, first order zero-crossing), tightness, mean speed and variability of spiral width. Linear mixed effect adjusted models comparing these indices and cross-validation were performed. Receiver operating characteristic analysis was applied to examine discriminative validity of combined indices.

**Results:**

All indices were significantly different between PD cases and controls, except for zero-crossing. A model using all indices had high discriminative validity (sensitivity = 0.86, specificity = 0.81). Discriminative validity was maintained in patients with early PD.

**Conclusion:**

Spiral analysis accurately discriminates subjects with PD and early PD from controls supporting a role as a promising quantitative biomarker. Further assessment is needed to determine whether spiral changes are PD specific compared with other disorders and if present in pre-clinical PD.

## Introduction

A discrete pre-clinical phase for Parkinson's disease (PD), characterized by early motor changes and non-motor manifestations, precedes the onset of diagnosable disease[1]. Identification of high-risk individuals, near the cusp of overt clinical PD, is important as it will promote better understanding of the disease pre-clinical evolution. Approaches to pre-clinical identification may facilitate enrollment of individuals at high risk for clinical PD and provide criteria for the enrollment of preventive intervention studies. Such measures may also provide objective, quantitative indices of motor decline, facilitating the conduct of secondary PD prevention studies[2]. While motor and non-motor biomarkers to detect at-risk individuals have been proposed, because PD is a motor disorder, very sensitive motor assessments are needed to assess the transition into clinical diseas*e*. Prior to assessing their utility in pre-clinical PD, such markers must first be validated to discern established PD from controls.

Motor rating scales, such as the Unified Parkinson’s Disease Rating Scale motor subscale[3] (UPDRS-III), the most widely used and accepted rating scale in PD, reflect symptom specific motor dysfunction and correlate with underlying neuropathology and treatment interventions. While easily performed, rating scales are examiner-dependent and relatively insensitive to subtle early disease[4]. Available methods to objectively assess human tremor and upper limb bradykinesia include accelerometer and gyroscopic systems[5, 6], electromagnetic or laser systems[7], the Purdue Pegboard[8], finger tapping[9] and digitizing tablets[10, 11]. The present study uses a digitizing tablet to facilitate computerized spiral analysis. This method quantifies upper limb motor function from hand drawn spirals using a digitizing tablet and writing pen connected to a computer. This method records the pen x and y positions, force and time data, without wires or other attachments. It has been used to evaluate many different types of neurological disorders (see review[12]) including multiple sclerosis[11], PD motor symptoms[13], essential tremor[14] and Niemann-Pick Disease[15]. Spiral degree of severity (DoS) has been validated with total motor UPDRS scores and correlates with disease severity[13].

However, depending on its specificity for disease, there is the potential that it could be capturing a late effect that is not abnormal in all PD subjects, and/or may be absent in early disease. Herein we extend the potential utility of spiral analysis in demonstrating that it has the capacity to distinguish established PD from controls, including early PD subjects, and thus warrants further assessment as a pre-clinical marker.

## Methods

### Subject selection and clinical ratings

PD subjects were recruited from parent studies of Biomarkers of PD at Mount Sinai Beth Israel Medical Center. A diagnostic checklist was completed and only those meeting strict PD diagnostic criteria[16] were included. Subjects were rated using the UPDRS by movement disorders neurologists and blinded to spiral performance. Unrelated unaffected control subjects were recruited from the pool of patient spouses visiting the movement disorders center of Columbia University Medical Center and BIMC, and through individuals enrolled in the Einstein Aging Study at the Albert Einstein College of Medicine. Controls were included if their age was between 40–89 years old and had no history of neurologic disorders, upper limb injuries, vision problems, or medication use for which tremor is a known side effect, and no history of rest or action tremor. The study was approved by the Institutional Review Boards (IRB) of Columbia University, Albert Einstein College of Medicine and BIMC and all participants gave informed written consent. Participating subjects completed a questionnaire that recorded demographic information as well as handedness, past medical history, and medication usage.

### Computerized spiral analysis

**Fig 1:**
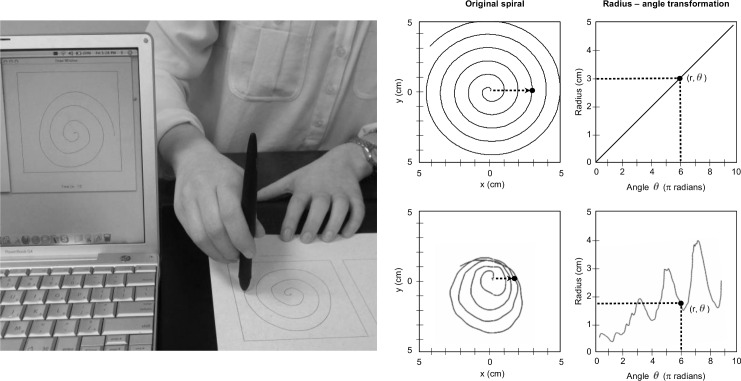
Digitized spiral analysis equipment and radius-angle transformation in spiral analysis. Digitized spiral analysis equipment (left) and radius-angle transformations from an ideal spiral (top row) and a PD patient (bottom row). The radius-angle transformation is the mathematical equivalent of “unraveling” the spiral such that the original two-dimensional graphic features, represented by (x, y) coordinates, are maintained with fidelity and expressed linearly in terms of (r, *θ*) coordinates. The example black dot shown is three full cycles (6 π radians) into each of the spiral drawings. In comparison to the ideal spiral straight transform, note the varying and irregular relationship between the radius and drawing cycles in the PD spiral.

Subjects were asked to draw ten Archimedean spirals with each hand inside a 10x10 cm square on 8.5x11 inch letter-size paper, using a wireless, inked writing pen on a 9x12 inch digitizing graphics tablet (Intuos 4, Wacom technology, Vancouver, WA) connected via standard USB to a computer using proprietary software written in Objective-C (Fig 1). The tablet had a resolution of 1,000 points/cm (2,540 points/inch), an accuracy of 0.025 cm (0.01 inches), and 256 levels of measurable pressure. Data were acquired at approximately 100 Hz. Subjects were shown an example spiral and were allowed to practice before spiral collection. All subjects used the same set-up, and were given the same instructions to sit with their shoulders parallel to the front edge of the tablet, not let the arms rest on the tablet, and draw spirals from the center outward. They were allowed to draw freely, to the extent that there were no constraints, attachments, or traceable templates, and they were asked to neither anchor nor rotate their drawing hand so that collection was standardized across all participants. The duration of test for training and the total of twenty spirals was approximately 20 minutes per subject. For those PD subjects on antiparkinsonian treatment, spirals were performed in the “on” medication state.

Data collected in the x, y, and z (pressure) axes and time provided virtual tri-axial recordings of spiral kinematics and dynamics. Features of spiral execution were expressed as mathematical indices using Matlab (The MathWorks, Natick, MA), as previously described[11]. Quantification of the spirals was based on radius-angle transformations of the two-dimensional spiral drawings which allowed for further computations of spiral analysis indices (S1 Material).

A matrix of 75 indices related to spiral execution including shape, loop tightness, tremors, speed and writing dynamics was calculated from the transformations. For this study, we investigated a subset of indices found clinically relevant in PD: overall DoS spiral execution, measures of shape and kinematics based on second order smoothness (2ndSm) and first order zero-crossing (1stZC), tightness (T), mean drawing speed (mSp) and a variability of loop-to-loop spiral width index (SWVI).

DoS was a unitless composite index that measured overall spiral execution and spatial irregularity[11], and correlates with worsening of total UPDRS motor scores [13]. It was designed as a computerized equivalent to the standard five-point clinical rating scale (0 to 4) of handwritten spirals where 0 to 1 = normal, 1 to 2 = mild, 2 to 3 = moderate, and 3 to 4 = severely abnormal. 2ndSm was a measure of spiral shape and curvature, defined as the derivative of how close the linear transformation of the spiral remained to its own mean[11]. It quantifies variation from ideal spiral shape. 1stZC measured how frequently the linear transform crossed its own mean. More frequent crossing indicated greater irregularity. Compared with 2ndSm, 1stZC was more sensitive to small or frequent drawing fluctuations. Both 2ndSm and 1stZC are unitless and mathematically describe spiral irregularity.

T (loops/cm) was the normalized number of turns of the spirals drawn over its total angular change within a 10x10 cm square. T was the correlate of clinical micrographia, and calculated as the average distance between consecutive spiral loops over all angles (in radians) divided by the maximum spiral radius (in cm). mSP (cm/sec) was calculated as the distance between all consecutive x, y points, averaged over the length of the spiral, divided by the average time between points. SWVI was a unitless kinematic measure of loop-to-loop spiral width variation with the oscillations of tremor removed, and is a correlate of ataxia[14]. Width variation highlights the fluctuations in spiral execution seen in patients with ataxia or erratic drawing (i.e., greater variability around an ideal trajectory). It is calculated as the coefficient of variation (ratio of the standard deviation to the mean) of the medians of spiral widths per angle over the entire 360° of each spiral loop. In addition, the variability between both hands (defined as the absolute value of the difference between dominant and non-dominant hand indices) was studied.

### Statistical methods

Analyses were conducted using the Statistical Analysis System SAS version 9.1 (SAS Institute, Cary, NC) and STATA12 (StataCorp, College Station, TX). Means and frequencies of demographic variables, UPDRS-III and spiral indices are described in Table 1. Exploratory univariate differences in the spiral indices between cases and controls and differences between dominant and non-dominant hand were analyzed using 2-tailed Student t-test, one-way ANOVA or non-parametric equivalent when appropriate. Linear mixed effect models adjusting for age, gender and handedness were used to compare the different indices between cases and controls. Models were performed including and excluding spiral severity. Receiver operating characteristic analysis using logistic models was applied to examine the discriminative ability of the combined indices. To assess the potential for over-fitting the model to the data, we used a three-fold, cross-validation study in which the data were randomly partitioned into three subsamples. The model was trained on two of the subsamples and validated using the remainder subsample. This was repeated three times. A separate analysis was performed in the subset of early PD (duration of ≤5 years).

**Table 1:** Demographic characteristics.

	Control Subjects	IPD	P-value
**Age** (years, mean±SD, range)	64.25±14.26 (40–94)	65.12±10.40 (34–88)	0.55
**Gender** (n,% Female)	91 (61%)	63 (46%)	0.01
**Handedness** (n, % Right-handed)	129 (86%)	118 (86%)	0.90
**Age at PD onset** (years, mean±SD)	—	58.50±10.90	—
**PD duration** (years, mean±SD)	—	7.25±4.73	—
**% Subjects on L-dopa** (n,%)	—	64 (46%)	—
**L-dopa dose** (median, IQR)	—	400 (200)	—
**Most affected side** (n, % Right side)	—	40 (28.78)	—
**Total UPDRS-III** (median, IQR)	—	13 (15)	—

## Results

**Fig 2:**
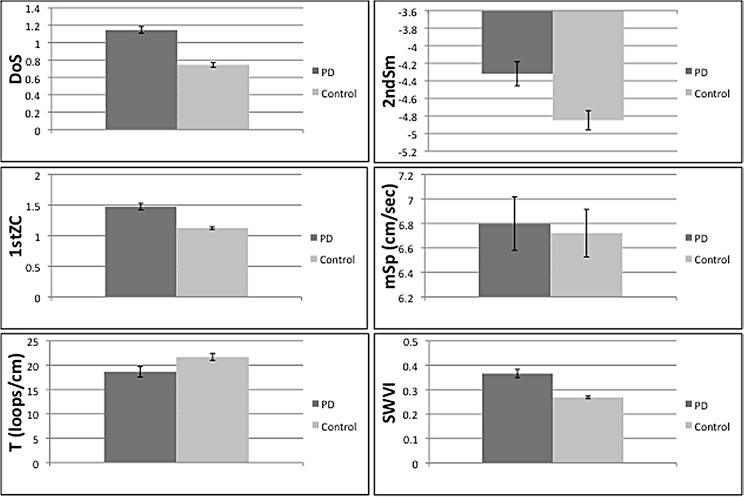
Spiral analysis indices in PD and controls. *p<0.05, mean spiral values presented except for SWVI (median).

138 subjects with PD and 150 unaffected controls were included in the study, including 50 PD subjects with disease duration of ≤5 years. Demographic and disease characteristics are described in Table 1. Groups did not differ for age and handedness; the male:female ratio was 75:63 among PD and 59:91 in controls. In the univariate analysis, all spiral indices except 1stZC were significantly different between cases and controls (Table 2, Fig 2). DoS in PD subjects was notably worse than controls (1.45 ± 0.45 vs. 0.74 ± 0.34, p<0.001). PD spirals had larger magnitude 2ndSm (-4.32 ± 1.60 vs. -4.85 ± 1.35, p = 0.002); PD spirals were performed slower (mSp 18.63 ± 12.71 vs. 21.69 ± 8.55 cm/s, p = 0.018) and were generally tighter (1.47 ± 0.63 vs. 1.12 ± 0.25, p<0.001). The median SWVI for the dominant-hand was also higher in PD than control subjects (0.33 vs. 0.26, p<0.001). The variability between both hands was significantly greater among cases for DoS, 2ndSm, T and SWVI.

**Table 2:** Dominant-hand Spiral Indices and Dominant–Non-dominant differences for PD Subjects and Controls.

**Index**			
	**Control**	**IPD**	**P-value**
DoS	0.74±0.34	1.45±0.45	<0.001
2ndSm	-4.85±1.35	-4.32±1.60	0.002
1stZC	6.72±2.38	6.80±2.56	0.790
mSp	21.69±8.55	18.63±12.71	0.018
T	1.12±0.25	1.47±0.63	<0.001
SWVI*	0.26 (0.07)	0.33 (0.13)	<0.001
Dominant–Non-dominant difference*			
DoS	0.25 (0.27)	0.37 (0.45)	0.020
2ndSm	0.77 (0.83)	0.93 (1.38)	0.006
1stZC	0.85 (1.20)	0.98 (1.36)	0.360
mSp	2.70 (3.50)	2.61 (4.47)	0.416
T	0.15 (0.18)	0.21 (0.30)	<0.001
SWVI	0.03 (0.05)	0.06 (0.08)	<0.001

*Median and IQR are presented. Mann-Whitney’s test was used for comparison.

PD subjects were classified according to motor subtypes into tremor-dominant (TD), intermediate or postural instability-gait disorder (PIGD) groups based on UPDRS-III scores[17]. Spiral indices did not vary according to motor subtype with the exception of SWVI, which was significantly higher in the PIGD group when compared to TD group (TD vs. PIGD p = 0.007).

A model using all the indices, including indices from both hands as well as variation between hands, had good classification accuracy in discriminating PD from unaffected control subjects (AUC = 0.875). The sensitivity and specificity from the optimal cut based on the Youden index were calculated at 0.860 and 0.812, respectively. In a cross-validation study, we divided the sample into three groups and examined the discriminative validity of cut-scores derived from two thirds of the sample on the remaining third for all possible combinations. The accuracy remained good from a three-fold cross-validation study (AUCs obtained were 0.845, 0.858 and 0.808, AUC_average_ = 0.837), which confirmed that these spiral indices are valuable markers in separating PD from controls. To assess the ability of spiral in distinguishing early PD (defined as disease duration either ≤5 years) from controls, we also performed ROC analysis restricted to early PD and controls and found that using spiral indices yielded good accuracy in diagnosing early PD from cross-validation studies (disease duration ≤5 years: AUC_average_ = 0.816; duration ≤4 years: AUC_average_ = 0.812).

## Discussion

Objective and reproducible metrics are needed when assessing subtle motor deficits in PD for accurate clinical evaluations and therapeutic interventions. There are still no good early PD biomarkers of motor function that are reliable and easy to obtain. To our knowledge, this is the largest study to date of upper limb motor control evaluating spiral drawing in PD. We found that spiral indices evaluating overall DoS, mSp, T, smoothness, and loop variability were significantly different between PD and controls (Table 2). These indices have been previously evaluated in PD and other movement disorders[13, 14]. In particular, in PD spirals, mSp was lower than in controls, and second-order smoothness, T and SWVI were also abnormal. Thus, PD spirals were tighter, performed more slowly and more irregularly than those drawn by controls. This is partly in concordance to previous studies. In one that utilized an optoelectronic system to quantitatively measure spiral execution motor features, spatial organization, size and regularity in PD subjects in the “on” medication state was very similar to that of controls, although their PD spirals were slower[18]. In another using a digitizing tablet, PD subjects drew spirals more slowly and were micrographic compared to normal controls[19].

Evaluation on PD subjects who were taking dopaminergic medications was performed in the parkinsonian “on” medication state. The decision to include only “on” medication spirals was to stress the potential of this technique to discern between milder forms of PD and controls, as treated PD subjects may closely resemble controls. Indeed, discrimination was present both in early PD and in the “on” state.

As PD usually manifests asymmetrically, a challenge for any motoric test involving the limbs in PD focuses on whether to capitalize on asymmetry and utilize data obtained from side-to-side differences, or to use the average of both sides. To capture subtle differences between dominant and non-dominant sides, which we would anticipate in early PD motor markers[20], we studied side-to-side variability between hands. This was defined as the absolute value of the difference between dominant and non-dominant hand indices. Similar differences are measured by the UPDRS-III[21], and may be consistent with the reported side of symptom onset[20]. We found significantly greater differences in the dominant non-dominant subtraction in all spiral indices except for 1stZC and mSp (Table 2). One possible explanation for the absence of mSp differences in a PD subject would be if the dominant, typically faster side, were affected first[21]. As there is an affinity for unilateral PD symptoms to occur most commonly in the dominant hand[20–22], there would be smaller differences between dominant and non-dominant mSp if the dominant side becomes slower first. An alternate explanation would be that by the time these PD subjects were evaluated, mSp may have decreased on both sides approximately by the same degree. As expected, the majority of our subjects reported right-hand dominance. 67% of PD subjects had asymmetric symptoms on their examination, defined as ≥2 UPDRS-III point difference between right and left sides[20]; however, only 32% of PD subjects had predominant symptoms on their dominant side vs. 34% in their non-dominant side and 34% had symmetric disease as defined (p = 0.40). Spiral indices were not significantly different between those with greater symptoms affecting the dominant vs. non-dominant sides with the exception of 2ndSm, which was greater in those individuals with worse symptoms affecting their non-dominant side (-4.74 vs. -4.00, p = 0.015). Dominant–non-dominant differences were significant only for degree of DoS and for 2ndSm. The difference in DoS and 2ndSm between dominant and non-dominant side were, perhaps as expected, also greater for those with worse symptoms affecting their non-dominant side (difference in DoS: 0.30 vs. 0.50, p = 0.001; difference in 2ndSm: 1.01 vs. 1.57, p = 0.02).

Motor subtype analysis into TD, intermediate and PIGD, showed that digitized spiral analysis had the capacity to discriminate between PD and controls in all motor subtypes, including non-TD PD, highlighting the sensitivity of the test to upper extremity bradykinesia and rigidity, in addition to its more traditional ability to detect tremor[10].

Multiple methods have been proposed to objectively assess and quantify upper extremity motor control in PD. Rating scales are widely accepted and used clinically such as the UPDRS[3]. Such scales reflect symptom specific motor dysfunction and correlate with underlying neuropathology and therapeutic interventions[23], and in some instances have been applied to mass screenings of parkinsonism[24]. Clinical rating scales, however, are examiner-dependent, insensitive to subtle disease and less reliable when administered by non-neurologists[4, 25]. The Purdue Pegboard is a simple, relatively inexpensive apparatus that has good test-retest reliability[8]. Finger tapping represents a simple task to quantitatively measure a motor deficit, and has been utilized to evaluate medication response in PD[26]. However, both Purdue Pegboard and finger tapping typically reflect speed and amplitude of movement without consideration for tremor, and finger tapping only addresses fine motor control of the hand without consideration of the proximal arm.

Digitized spiral analysis as presented in this study may provide advantages compared to other proposed methods. A portable wireless motion sensor device, Kinesia (Cleveland Medical Devices Inc., Cleveland, OH) provides quantitative motor scores, and correlates to clinical rating scales[27, 28]. However, the set guided motor, tremor and bradykinesia tasks as well as the wearable hardware may limit the free movement of the hand. A similar version of digitized spiral analysis evaluated tremor intensity changes before and after ethanol ingestion in subjects with alcohol-responsive essential tremor, and showed adequate validity, reliability and sensitivity to reflect such changes[29]. The subjects drew spirals between lines, forcing spatial consistency across different spirals and might have restricted speed and other kinematic measures. The type of upper limb motoric assessment presented in our study is safe, inexpensive, relatively fast to perform, non-invasive, and does not require the use of wires or other attachments that may limit upper limit movement. Information regarding time, pressure, and much more carefully quantified data is available with a digitizing tablet than with, for example, measurement of 2-D pen and paper spirals without other quantification. Spiral analysis may have even better predictive capacity when combined with one of these other methods, or in combination with gait or lower leg assessment.

Subtle writing changes may occur early in the course of PD and limit activities. This was noted even in the early 19^th^ century when statesman and educational reformer, von Humboldt, recorded his small writing in a letter in 1823, and it was not until 1828 that he noted hand tremor and difficulty with repetitive movements[30]. For individuals who suffer from concomitant memory loss, difficulty with writing impedes a useful technique for reminding and writing things down. Therefore, there is both clinical relevance but ecologic utility in evaluating spiral analysis.

There are some limitations to our study. Spiral analysis with the digitizing tablet is a neurophysiological and psychophysical test with a critical need for the proper conditions and set-up, so collection may be problematic for unsupervised home use. While the instructions were limited, all data were collected by an examiner who started and stopped the task, and directed the subject to the next spiral. Although digitized spirals are easily and quickly collected in the clinical setting, analysis is off-line and requires technical sophistication and competence. Also, quantitative motor measurements are limited to the upper extremity. Nevertheless, PD commonly affects the upper limb and an important amount of disability is derived from arm/hand dysfunction. Advantages of this technique are that the subject freely draws spirals without attachments, wires or the restrictions involved when tracing. Also, both distal and proximal arm movements are involved, facilitating study of the entire upper limb, and not just the hand. Evaluations in the "on" and "off" state to determine the effect of levodopa on spiral indices are of interest and currently underway. Recruitment of opposite sex spouses poses the limitation of gender disbalance between cases and controls. However, men and women do not differ in spiral performance,[31] and we performed a sensitivity analysis limited men alone (S1 Table), we found the same direction and significance of results. Finally, while it has been demonstrated that spiral analysis separates controls from other disorders[14, 29], no direct comparisons were performed between PD and other tremor disorders, and further study comparing essential tremor and PD-mimics to PD is warranted.

Together with others studies demonstrating that digitized spiral analysis detects changes in the unaffected side that were not clinically measurable by the UPDRS-III in very early and unilateral PD, and can distinguish early PD from controls, suggests that digitized spiral analysis warrants further evaluation as a potential marker to detect PD at its earliest, pre-clinical stages.

## Supporting Information

S1 Material Definitions for Digitized Spiral Analysis. (DOCX)

S1 Table Dominant-hand Spiral Indices and Dominant-Nondominant Differences for PD Subjects and Controls by Gender, Sensitivity Analysis Limited to Men. (DOCX)
