# Supplementary material for: Digitized Spiral Drawing: A Possible Biomarker for Early Parkinson’s Disease
Source: PLoS One. 2016 Oct 12;11(10):e0162799. doi: 10.1371/journal.pone.0162799 (PMC5061372; doi:10.1371/journal.pone.0162799)
Supplement: S1 Material — (DOCX) [file pone.0162799.s001.docx]

**SUPPLEMENTARY MATERIAL**

**S1 Material. Definitions for Digitized Spiral Analysis**

The **radius-angle transformation** was derived from spiral (x, y) data points, and defined as x = *r* sin θ + x_0_, y = *r* cos θ + y_0_ where (x_0_, y_0_) was the spiral center. The radius *r* = and (*r*, θ) was its counterpart in the polar expression. This simplifies to the polynomial relationship: θ = *a r* , where *a* is a positive constant.

**Degree of spiral drawing severity (DoS):**

DoS was a unitless composite index that measured overall spiral execution and spatial irregularity[[11](#_ENREF_11)], and correlates with worsening of total UPDRS motor scores[[13](#_ENREF_13)]. It was designed as a computerized equivalent to the standard five-point clinical rating scale (0 to 4) of handwritten spirals where 0 to 1 = normal, 1 to 2 = mild, 2 to 3 = moderate, and 3 to 4 = severely abnormal.

The formula (not in the text) is:

DoS = 0.4615 * I_1_ + 0.0544 * I_5_ - 0.2331 * I_12_ - 0.0726 * I_22_ - 0.001 * I_5_^2^ + 0.2539 * I_1_*I_2_ + 1.3668

where I_1_ = first order smoothness, I_2_ = second order smoothness, I_5_ = second order zero crossing

**Second order smoothness (2ndSm)**

2ndSm was a measure of spiral shape and curvature, defined as the derivative of how close the linear transformation of the spiral remained to its own mean[[11](#_ENREF_11)]. It quantifies variation from ideal spiral shape.

The formula (not in the text) is:

2Sm = $\ln\left[\frac{1}{\Theta}\sum\left(\frac{\Delta\frac{\Delta r}{\Delta\theta}}{\Delta\theta}-d\overline{r}_{\theta}\right)^{2}\right]$

where $\Theta$ = Total angle over which the spiral is drawn

$d\overline{r}_{\theta}$ = The derivative of the rms $\Delta r/\Delta\theta$ value

**First order zero crossing (1stZC)**

1stZC measured how frequently the linear transform crossed its own mean. More frequent crossing indicated greater irregularity. Compared with 2ndSm, 1stZC was more sensitive to small or frequent drawing fluctuations.

Both 2ndSm and 1stZC are unitless and mathematically describe spiral irregularity.

The formula (not in the text) is:

where J = Total number of data points in the series of acquired data

j = A specific data point in the series (i.e. data point #5)

= Sum from first data point acquired (j=1) to the 2nd to last data point (J-1).

$\overline{r_{\theta}}$= The rms value of $\frac{\Delta r}{\Delta\theta}$

Note the “sign” function, which is essential to this calculation.

The output of the sign function has three values: 1, 0, or -1, depending on whether the

value “x” (comprised of the terms $\mathop{\left(\frac{\Delta r}{\Delta\theta}\right)}\limits_{j+1\_or\_j} -\left(\frac{\Delta r}{\Delta\theta}\right)_{\overline{r_{\theta}}}$in the equation) is greater than, equal to, or less than zero:

sign(x) = 1, if x>0

sign(x) = 0, if x=0

sign(x) = -1, if x<0

**Tightness (T)**

T (loops/cm) was the normalized number of turns of the spirals drawn over its total angular change within a 10x10 cm square. T was the correlate of clinical micrographia, and calculated as the average distance between consecutive spiral loops over all angles (in radians) divided by the maximum spiral radius (in cm).

The formula (not in the text) is:

Tightness (spiral loops/cm of radius) = $\frac{\frac{\Theta_{\begin{matrix} actual \\ spiral \end{matrix}}}{R}_{\begin{matrix} actual \\ spiral \end{matrix}}}{\frac{2\pi}{cm}}$

where $\Theta$ (in radians) = the total angle of the spiral

net radius R (in cm) = largest radius attained at the outermost loop of the spiral

**Mean drawing speed (mSp)**

mSP (cm/sec) was calculated as the distance between all consecutive x, y points, averaged over the length of the spiral, divided by the average time between points.

**Variability of spiral width (SWVI).**

SWVI was a unitless kinematic measure of loop-to-loop spiral width variation with the oscillations of tremor removed, and is a correlate of ataxia [[14](#_ENREF_14)]. Width variation highlights the fluctuations in spiral execution seen in patients with ataxia or erratic drawing (i.e., greater variability around an ideal trajectory). It is calculated as the coefficient of variation (ratio of the standard deviation to the mean) of the medians of spiral widths per angle over the entire 360° of each spiral loop.
