# Supplementary material for: Digitized Spiral Drawing: A Possible Biomarker for Early Parkinson’s Disease
Source: PLoS One. 2016 Oct 12;11(10):e0162799. doi: 10.1371/journal.pone.0162799 (PMC5061372; doi:10.1371/journal.pone.0162799)
Supplement: S1 Table — (DOCX) [file pone.0162799.s002.docx]

**S1 Table.** Dominant-hand Spiral Indices and Dominant – Non-dominant differences for PD Subjects and Controls by Gender, sensitivity analysis *limited to men*

| **Index** | | | |
| --- | --- | --- | --- |
|  | **Control (n=59)** | **IPD (n=75)** | **P-value** |
| DoS | 0.68±0.31 | 1.22±0.43 | <0.001 |
| 2ndSm | -5.02±1.26 | -3.99±1.62 | <0.001 |
| 1stZC | 6.51±2.15 | 7.18±3.06 | 0.157 |
| mSp | 21.98±7.63 | 16.65±11.60 | 0.003 |
| T | 1.10±0.24 | 1.55±0.74 | <0.001 |
| SWVI* | 0.26 (0.06) | 0.31 (0.14) | <0.001 |
|  |  |  |  |
| **Dominant – Non-dominant difference*** | | | |
| DoS | 0.21 (0.27) | 0.27 (0.40) | 0.184 |
| 2ndSm | 0.61 (0.72) | 0.58 (1.15) | 0.630 |
| 1stZC | 0.71 (1.17) | 1.24 (1.86) | 0.012 |
| mSp | 3.01 (4.19) | 2.38 (3.04) | 0.403 |
| T | 0.16 (0.18) | 0.24 (0.32) | 0.002 |
| SWVI | 0.03 (0.04) | 0.06 (0.05) | <0.001 |

*Median and IQR are presented. Mann-Whitney’s test was used for comparison
